# Vitamin D Deficiency Is Frequent in Patients with Rapidly Destructive Osteoarthritis—Data from a Single-Center Analysis

**DOI:** 10.3390/jcm13051296

**Published:** 2024-02-25

**Authors:** Konstantin Horas, Gerrit Maier, Maximilian Rudert, Axel Jakuscheit, Manuel Weißenberger, Ioannis Stratos, Tizian Heinz, Dominik Rak, Philip Mark Anderson, Jörg Arnholdt

**Affiliations:** 1 Department of Orthopaedic Surgery, Koenig-Ludwig-Haus, University of Wuerzburg, 97074 Wuerzburg, Germany; 2 Department of Orthopaedic Surgery, Pius-Hospital, Carl-von-Ossietzky-University, 26121 Oldenburg, Germany; 3 Orthopaedic Surgery Centre Wuerzburg (OCW), 97070 Wuerzburg, Germany; 4 Department of Orthopaedics and Trauma Surgery, Musculoskeletal University Center Munich (MUM), LMU University Hospital, 81377 Munich, Germany

**Keywords:** vitamin D deficiency, rapidly destructive osteoarthritis, rapidly progressive osteoarthritis

## Abstract

Background: Rapidly destructive osteoarthritis (RDO) of the hip joint is characterised by the rapid destruction of the femoral head with or without acetabular involvement. There has been increasing interest in this disease over the past years; however, the entity is still poorly understood, and its pathophysiology remains unknown. Yet, there is ample evidence today that increased bone metabolism might play a role in the onset and progression of the disease. Vitamin D is of utmost importance to maintain a balanced bone metabolism. However, whether vitamin D deficiency is involved in disease development remains to be elucidated. Further, the vitamin D status of patients with RDO has not yet been analysed. For this reason, the objective of this study was to assess the vitamin D status of patients with RDO. Moreover, the aim was to clarify whether there is a difference in the vitamin D status of patients with RDO compared with patients with primary osteoarthritis (OA). Methods: In this single-centre analysis, the 25(OH)D, PTH, and calcium levels of 29 patients who presented with RDO between 2020 and 2022 were assessed. Results: Altogether, 97% of patients (28/29) were vitamin D deficient, a further 3% (1/29) were vitamin D insufficient, and not a single patient presented with a sufficient vitamin D status. Notably, the vitamin D levels of RDO patients (mean = 11.04 ng/mL) were significantly lower than the vitamin D levels of patients with OA (mean = 22.16 ng/mL, *p* = 0.001). Conclusion: In conclusion, we found a widespread and high rate of vitamin D deficiency in patients with RDO. Hence, we believe that 25(OH)D status should routinely be analysed in these patients.

## 1. Introduction

**Figure 1:**
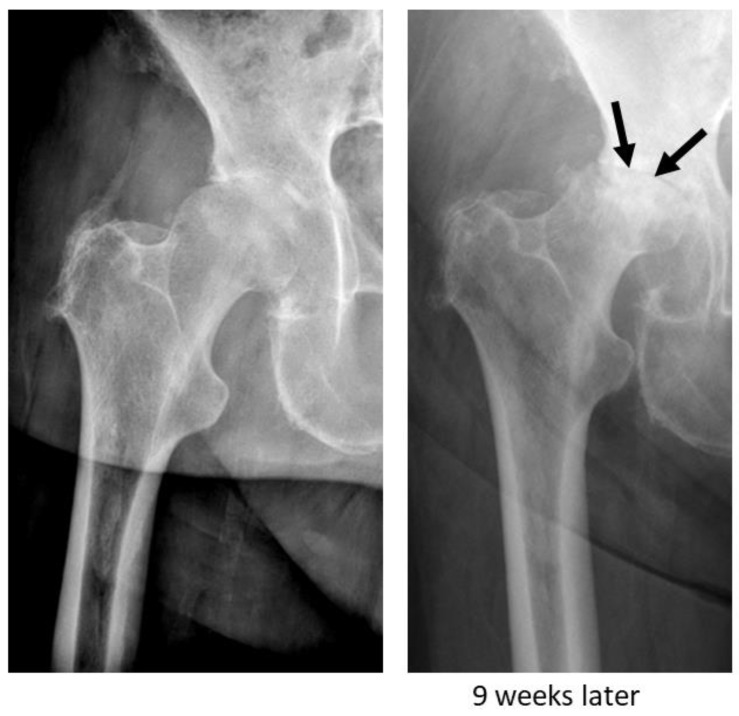
X-ray of a right hip joint (ap) of a 77-year-old female patient with osteoarthritis (**left**) and X-ray 9 weeks later showing rapidly destructive osteoarthritis (arrow) (**right**). The patient had no known risk factors for osteoporosis.

Rapidly destructive osteoarthritis (RDO) of the hip is a clinical entity that was first described in 1957 by Forestier [1]. It is characterised by a rapid destruction of the hip joint that often occurs within months of the onset of symptoms [2]. While initial radiographs oftentimes show rather mild osteoarthritis of the hip joint, follow-up imaging obtained a few months or even weeks later demonstrates the progressive destruction of the femoral head, with or without acetabular involvement [3]. In some cases, the almost complete disappearance of the femoral head within a few months can be observed (Figure 1) [4]. Generally, either the right or the left hip joint is affected, and it is mostly seen in elderly females aged between 70 and 75 years [5]. However, there are also reports of bilateral appearance. Clinically, patients present with acute severe and rapidly worsening hip pain [4,5]. Today, multiple descriptive terms for this condition, such as rapidly destructive osteoarthritis (RDO), rapidly progressive coxarthrosis, Postel’s osteoarthritis, and rapidly progressive hip disease, have been proposed [3]. Furthermore, there have been several attempts to classify this pathological condition depending on the amount of bone loss and the time period over which this takes place [3,6,7]. However, it remains difficult to diagnose, as there is no single pathognomonic diagnostic finding and no specific symptom that allows for clear differentiation between RDO and primary osteoarthritis. Furthermore, its oftentimes unpredictable behaviour makes it even more challenging for physicians to identify and estimate disease progression. Thus, in many cases, surgical intervention is needed for advanced-stage RDO, with sometimes considerable acetabular bone loss. This in turn might result in the use of bigger and more complex implants to achieve appropriate surgical treatment [5,8]. Moreover, conservative treatment does not seem to provide symptomatic relief or slow its progression. For this reason, surgical treatment appears to be the only appropriate therapeutic option. The reported incidence of RDO ranges from around 3 to 15.7% [5,9,10,11]. Due to the ageing population, the number of people diagnosed with RDO is estimated to increase [8]. The pathophysiology of RDO is mostly unclear, and so far, it remains a poorly understood disease [3,7,8]. However, there is now increasing evidence that impaired bone metabolism and osteoclast activation play a role in the onset and progression [12,13]. It is also conceivable that osteoporosis (OP) has an impact on the development of RDO. OP is frequent in elderly people and characterised by increased osteoclastic bone resorption that is not compensated by osteoblastic bone formation. These properties are similar to what is found in patients with RDO; thus, pre-existing OP might provide a potential rationale for disease development. At present, it remains unclear whether there is a relationship between OP and RDO.

Vitamin D is crucial in the maintenance of healthy bone metabolism [14]. Its classical function is the regulation of calcium and phosphate homeostasis; thus, it impacts bone remodelling. The process of bone remodelling is a life-long and susceptible continuum aiming to break down old mechanically unnecessary bone and replace the removed tissue with an equal amount of newly formed bone [15]. Under physiological conditions, resident osteoclasts and osteoblasts continuously remove and replace bone in a well-orchestrated manner [16]. Persistently low vitamin D status leads to an increase in bone turnover via the upregulation of parathyroid hormone (PTH). PTH is a peptide hormone that binds to osteoblasts, which in turn leads to the activation of osteoclasts via the OPG–RANKL–RANK system [17]. Thus, PTH has a stimulatory effect on bone resorption, as it indirectly increases the number and activity of osteoclasts. Vitamin D deficiency is a global health concern that is estimated to afflict over one billion people globally [15]. Vitamin D deficiency is also frequent in orthopaedic patients [18]. Furthermore, its prevalence increases with age, thus coinciding with the rise in the incidence of RDO [19].

Collectively, there is mounting evidence that altered bone metabolism plays a role in RDO. However, extensive studies and robust data on bone metabolism in these patients are scarce. As such, additional studies are of utmost importance to further elucidate a potential association. We hypothesised that patients with RDO have low vitamin D status, which results in altered bone turnover. Consequently, the bone is at higher risk for the development and progression of RDO than the bone in patients with regular vitamin D status. For this reason, the objective of this study was to analyse the vitamin D status of patients with RDO. In particular, our aim was to analyse how vitamin D status varies between patients with RDO and patients with primary osteoarthritis (OA).

## 2. Patients and Methods

Patient selection: Between January 2020 and December 2022, the serum 25(OH)D (the circulating form of vitamin D, a robust and reliable marker of vitamin D status), PTH, and calcium levels of 29 patients who presented with RDO were measured at the Department of Orthopaedics, Koenig-Ludwig-Haus, University of Wuerzburg, Germany (49.76° N latitude). To confirm the diagnosis, every patient underwent a thorough preoperative workup to exclude any other diseases that could be responsible for joint destruction. Radiographic evaluation was performed by two experienced reviewers. Patients were excluded from the study if no previous X-rays or magnetic resonance imaging (MRI) for comparison were available. Moreover, patients who presented with the destruction of the hip joint due to any other known reason, such as avascular necrosis of the femoral head or secondary osteoarthritis, were excluded. Further exclusion criteria were known diseases that potentially impact vitamin D status, such as general bone metabolism disorders, hypo-/hypercalcaemia, and hypo-/hyperparathyroidism. Additionally, patients who reported routine intake of vitamin D supplements or drugs specifically affecting bone health, such as PTH analogues, bisphosphonates, and RANKL inhibitors, were excluded. A previously generated control group of 118 otherwise healthy patients with primary osteoarthritis of the hip joint was used for comparison. In total, 29 patients who presented with RDO were enrolled in this study; 19 patients were female, and 10 patients were male, with a cumulative mean age of 70.17 years (range 47–84 years).

Radiographic assessment: For the diagnosis of RDO, there is no generally accepted period between the first appearance of symptoms to the radiologically confirmed destruction of the hip joint. We decided to include all patients in whom the femoral head was degraded by at least one-third of its initial size within six months. Measurements were conducted using radiographic imaging (DeepUnity Review, DH Healthcare GmbH, Bonn, Germany).

Serum measurements: All experiments were conducted in accordance with the guidelines of the Committee of Medical Ethics (University of Wuerzburg, Germany, ethics number 186/18-am) and in accordance with the World Medical Association Declaration of Helsinki. Approval for the collection of venous blood samples and testing for serum 25(OH)D, PTH, and calcium was obtained from every patient. Blood samples were generally collected at the first time of diagnosis. A standardised method for serum measurements using the Cobas^®^ 25-Hydroxyvitamin D Assay (Vitamin D Total) and the Elecsys PTH (1–84) assay for the Cobas^®^ e 411 Analyzer (Roche Diagnostics, Mannheim, Germany) was applied. All laboratory results were collected using a retrospective chart review. Moreover, we screened patients for risk factors for osteoporosis, assessing comorbidities and routine medication intake. Hence, we used the guidelines of the German Osteology Society (Dachverband Osteologie, DVO).

Data evaluation: To date, there is no international consensus on what serum 25(OH)D level can be regarded as insufficient [20]. For data analysis and interpretation, we followed the guidelines of the Endocrine Society defining vitamin D deficiency as a 25(OH)D level of less than 20 ng/mL (50 nmol/L) and vitamin D insufficiency as a 25(OH) D level between 20 and 29 ng/mL (50–72.5 nmol/L) [20]. Consequently, serum 25(OH)D levels greater than or equal to 30 ng/mL (75 nmol/L) were considered vitamin D sufficient [20]. All patients were included in the statistical analysis and grouped according to gender, age, season, and affected side. The Kolmogorov–Smirnov test and Shapiro–Wilk test were used to check for the normal distribution of the data. For numerical data (e.g., serum vitamin D, PTH, and calcium levels) the Mann–Whitney U test or independent Student’s *t*-test were used. Nominal data were evaluated using the Chi-square test. Differences in serum vitamin D levels between subgroups were evaluated using the Kruskal–Wallis test. The level of statistical significance was set at *p* < 0.05.

## 3. Results

**Figure 2:**
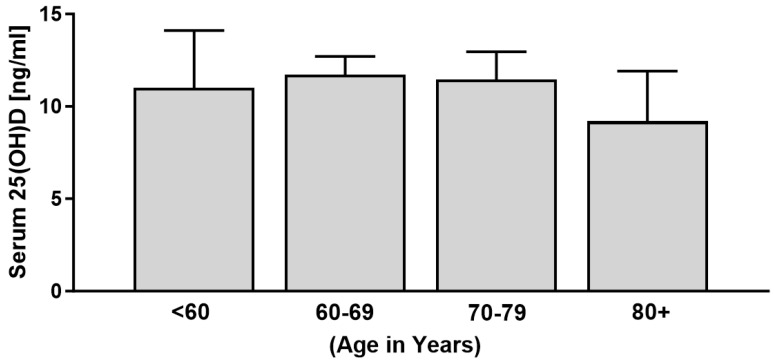
Serum vitamin D levels of patients with RDO grouped according to age (n = 29).

All patients (29/29) presented with low vitamin D levels, with a total mean 25(OH)D level of 11.04 ng/mL (27.6 nmol/L). Particularly, 97% of patients (28/29) were vitamin D deficient, a further 3% (1/29) were vitamin D insufficient, and not a single patient presented with vitamin D levels greater than or equal to 30 ng/mL (75 nmol/L) that could be regarded as sufficient. For the comparison of age groups, we further subdivided the cohort according to age into under 60, 60–69, 70–79, and over 80 years old. Vitamin D status was low in all age groups but particularly low in patients aged 80 years or older (Figure 2). However, there was no statistical difference regarding age group and vitamin D status (X^2^ (df = 3, N = 29) = 1.60, *p* = 0.66).

**Figure 3:**
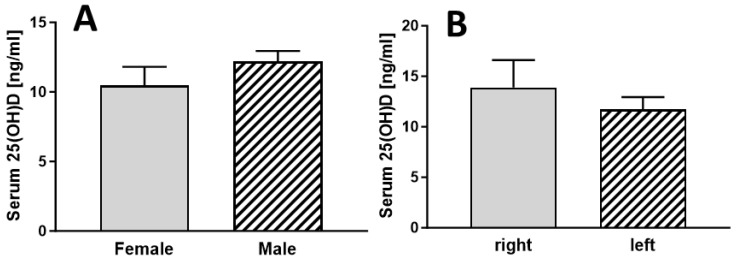
Serum vitamin D levels of patients with RDO grouped according to gender ((**A**), n =19 for female and n = 10 for male) and affected side ((**B**), n = 19 for right and n = 10 for left side).

There were no statistically significant differences between females and males (Figure 3A) or between whether the right or left side was affected (Figure 3B).

**Figure 4:**
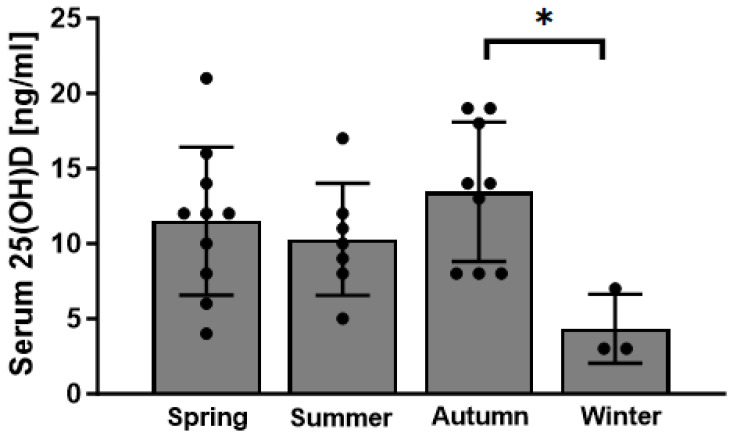
Serum vitamin D (25(OH)D) levels of patients with RDO in correlation with the season (n = 10 for spring (March–May); n = 7 for summer (June–August); n = 9 for autumn (September–November); n = 3 for winter (December–February) * *p* < 0.05).

Most of the body’s vitamin D is produced in the skin in a UVB-dependent manner. As such, the production of vitamin D depends not only on the capacity of the skin to do so but also on daily sun exposure. To determine whether there were differences in vitamin D levels depending on the season, we subdivided cohorts into four groups according to the mean sunshine hours in Germany (spring = March–May; summer = June–August; autumn = September–November; winter = December–February). Serum vitamin D levels were low in general; however, they were particularly low in wintertime (Figure 4). A statistical difference in serum vitamin D levels was found between autumn and winter (*p* = 0.04) (Figure 4).

**Figure 5:**
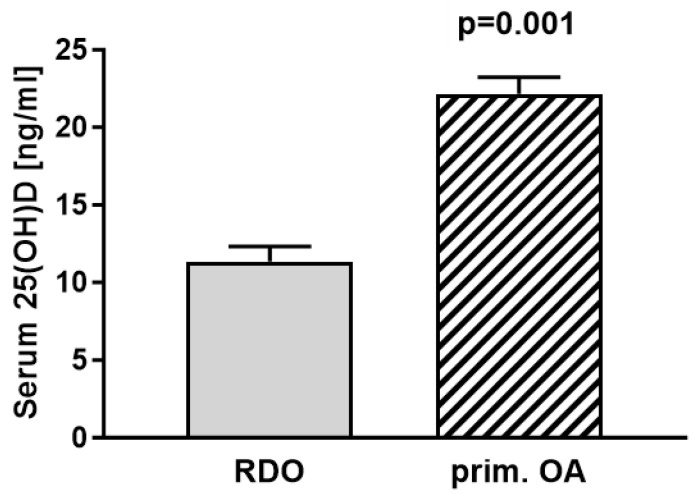
Comparing serum vitamin D (25(OH)D) levels of patients with RDO (mean 11.04 ng/mL (27.6 nmol/L); n = 29) to patients with primary osteoarthritis (mean 22.16 ng/mL (55.4 nmol/L); n = 118) revealed significant differences between groups (*p* = 0.001).

Statistical analyses of the vitamin D levels of RDO patients (mean = 11.04 ng/mL) compared with the vitamin D levels of patients with primary osteoarthritis (mean = 22.16 ng/mL) showed significantly lower vitamin D levels in patients with RDO (*p* = 0.001) (Figure 5).

Altogether, 18 patients reported routine intake of medication that is considered a risk factor for osteoporosis (according to guidelines of the DVO). Among the most frequent were regular intake of proton-pump inhibitors (PPIs), depression medicines or opioids. Comorbidities that are associated with osteoporosis were present in 10 patients. The three most frequent were nicotine dependence, diabetes mellitus, and depression. Due to the small sample size, the interaction between vitamin D, calcium, and PTH levels and comorbidities or oral medication was not evaluated. Hence, we omitted further statistical tests of significance.

## 4. Discussion

The findings from the current study show a high rate of vitamin D deficiency in patients with RDO. It is a hitherto unknown aspect of RDO that vitamin D deficiency might be a potential risk factor for the onset and progress of the disease. Since the cause of RDO is still unknown, finding an appropriate treatment to slow down progression is challenging and remains of great importance [5]. Recent findings point to an association between RDO and impaired bone metabolism [21]. For example, Ogawa et al. compared histological and functional findings in RDO with those in slowly progressive osteoarthritis (OA) to investigate whether osteoclasts contribute to the extensive bone destruction observed [21]. They found that mature activated osteoclasts existed only in the synovium of RDO and not in the OA synovium, which pointed to an association with osteoclastogenesis [21]. These results are supported by a study from Yamakawa et al., who also found increased osteoclast numbers on the bone surfaces of rapidly progressive arthritic hips [22]. Mitrovic et al. analysed the synovial fluid, articular cartilage, and bone changes in ten femoral heads of patients with rapidly destructive arthropathy [23]. They identified bone marrow atrophy and fibrosis as well as intense bone remodelling in RDO samples. In yet another study, Yasuda et al. analysed bone turnover markers in early-stage rapidly progressive osteoarthritis of the hip [24]. Interestingly, they found that bone resorption markers, such as tartrate-resistant acid phosphatase-5b (TRAP-5b) and bone-specific alkaline phosphatase (BAP) were significantly increased in RDO patients [24]. This is in line with previous studies and our own findings, as increased bone resorption markers are regularly present in patients with vitamin D deficiency [25,26,27]. Collectively, there is evidence that alterations in bone turnover and increased bone remodelling are involved in RDO. However, other studies have failed to support such associations. For example, Okano et al. investigated bone mineral density in 17 patients with RDO and compared it to the BMD of 75 patients with osteoarthritis of the hip [28]. No significant differences were observed in the BMD of the lumbar spine, ultradistal radius, mid-radius, or calcaneus between groups. Nonetheless, this study only looked at the bone at the macro level and not at the hip joint, which is the area of interest. Other authors have not seen impaired bone turnover as a main factor that drives the disease and rather believe in a relationship between RDO and increased inflammation or subchondral insufficiency fractures [4,8].

Healthy bone is a *conditio sine qua non* for successful cementless total hip arthroplasty. If the bone quality is not sufficient to allow for press-fit implantation and natural bone growth to bind the implant to the bone, cemented hip arthroplasty is generally used to fix the implant surface to the bone. This is often the case in osteopenic or osteoporotic patients who have thin, weak, and brittle bones [29]. Furthermore, many orthopaedic surgeons generally prefer hybrid or fully cemented hip arthroplasty in elderly patients [29]. Since RDO patients often suffer from severe pain, cementless, hybrid, or fully cemented joint arthroplasty is commonly performed. However, as the cause of RDO is still unknown, there are presently no generally accepted guidelines for the treatment of this disease. In particular, it is not generally recommended to use hybrid or fully cemented arthroplasty in these patients. The results from the current study clearly demonstrate that patients with RDO have a high risk of vitamin D deficiency. Furthermore, many patients in our study had additional risk factors (either via routine medication or comorbidities) for osteoporosis. Thus, orthopaedic surgeons should consider the use of hybrid hip arthroplasty, especially as patients are oftentimes 70 years of age or older [20]. Independent of these considerations, clinicians should be aware that patients with RDO not only often present with low vitamin D levels but also commonly have risk factors for osteoporosis. This is of clinical relevance, as surveys have shown that orthopaedic surgeons adapt their surgical plan and implant design according to previously known comorbidities affecting bone health and BMD, such as osteoporosis [30]. Moreover, this might also impact the outcomes for patients [20]. However, there is currently a paucity of data on the long-term outcomes for RDO patients, and more well-constructed studies are therefore required [5].

Vitamin D deficiency is frequent and has become a worldwide problem of a considerable extent [31]. Over the past decades, research on vitamin D has been progressively more popular, which has led to a plethora of studies investigating potential health-beneficial effects of vitamin D [15,32,33,34]. Consequently, it is now known that vitamin D impacts a multitude of diseases, including many orthopaedic disorders [16,35,36,37,38]. Thus, it is conceivable that vitamin D deficiency might also play a role in the onset and progression of RDO. 

To the best of our knowledge, this is the first report of a study analysing the vitamin D status of patients with RDO. Our findings clearly indicate that vitamin D deficiency is common in these patients. Although it remains unclear whether vitamin D plays a role in the onset or progression of the disease, our results evidently indicate that there might be an association between these two apparently independent pathologies. Nonetheless, the reported association does not prove any causal relationship, and further studies and especially robust data are needed to confirm these results.

The data shown in this study represent single-centre patient data from the geographical localisation of Wuerzburg (Bavaria, Germany (49°47′ N latitude)). For this reason, the patient data can only be compared with serum 25(OH)D levels of patients living at latitudes comparable to those of Wuerzburg (e.g., Paris (48°51′ N), Seattle (47°37′ N), Calgary (51°07′ N), Vancouver (49°15′ N), and Kyiv (50°27′ N)). However, comparing the vitamin D levels of RDO patients with those of patients with OA in our control group (mean 11.04 ng/mL versus 22.16 ng/mL) showed highly significant differences between groups (*p* = 0.001). Furthermore, comparing data from the current study to those on the vitamin D status of the general population in Germany, striking differences remain [39,40]. The seasonal variations found in our study are in line with those of previous reports, and it is not surprising that vitamin D status was lowest in winter and spring [20,36]. We are also aware that the significance of our findings is limited due to a rather small patient sample size, which is attributable to the rare entity of this disease. Furthermore, we are aware that there might be selection bias in the control group. Therefore, the results have to be interpreted in a critical way before drawing any conclusions. Another limitation is that only severe and advanced-stage RDO patients were included in this study (all cases were grade III according to Zazgyva et al. [41]). For this reason, early-stage patients are clearly underrepresented in this study.

In summary, the considerably low vitamin D levels found in patients with RDO in this study provide evidence that vitamin D deficiency might play a role in the onset and progression of the disease. Hence, there should be an increased awareness among physicians to assess the vitamin D status of patients with RDO. Yet, the question as to whether patients with low vitamin D levels in general are more prone to develop RDO and thus would profit from vitamin D supplementation remains unanswered. To elucidate the mechanism, in-depth analyses of impaired bone metabolism in these patients must be performed in future studies. These should include not only the measurement of markers of bone remodelling but also histological analyses of affected bones. Finally, many open questions remain, which will need to be addressed by future research.

## 5. Conclusions

This single-centre analysis identified a concerning rate of vitamin D deficiency in patients with RDO. Notably, the vitamin D levels of patients with RDO were significantly lower than those of patients with primary OA of the hip joint. Furthermore, several RDO patients exhibited risk factors for osteoporosis. Hence, we recommend that vitamin D levels should routinely be examined in patients with RDO.

## Data Availability

The data presented in this study are available on request from the corresponding author.
